# Trans fatty acid intake increases likelihood of dyslipidemia especially among individuals with higher saturated fat consumption

**DOI:** 10.31083/j.rcm2304130

**Published:** 2022-04-07

**Authors:** Emmanuella Magriplis, Georgios Marakis, Sotiria Kotopoulou, Androniki Naska, George Michas, Renata Micha, Demosthenes Panagiotakos, Antonis Zampelas

**Affiliations:** ^1^Department of Food Science and Human Nutrition, Agricultural University of Athens, 11855 Athens, Greece; ^2^Hellenic Food Authority, 11526 Athens, Greece; ^3^Department of Hygiene, Epidemiology and Medical Statistics, School of Medicine, National and Kapodistrian University of Athens, 11527 Athens, Greece; ^4^Department of Food Science & Human Nutrition, University of Thessaly, 43100 Karditsa, Greece; ^5^Friedman School of Nutrition Science and Policy, Tufts University, Boston, MA 02111, USA; ^6^Department of Nutrition and Dietetics, School of Health Science and Education Harokopio University, 17676 Athens, Greece

**Keywords:** trans fatty acid intake, dyslipidemia, dietary intake, saturated fat intake, cardiovascular disease, food contribution

## Abstract

**Background:**

Evidence points to adverse effects of trans fatty acids (TFA) on health. The aim of this study was to estimate total TFA intake, evaluate major food contributors and its effect on dyslipidemia.

**Methods:**

A total of 3537 adults (48.3% males) were included. Total TFA intake was assessed using two 24-hour dietary recalls. Foods were categorized into specific food groups. Adjusted Logistic Regression analysis was performed to assess the likelihood of dyslipidemia by tertile of TFA aand Saturated Fatty Acid (SFA) level.

**Results:**

Median TFA intake was 0.53% of energy (from 0.34 to 0.81) ranging from 0.27 (Q1) to 0.95 (Q3) (*p *< 0.001, for trend), and 16% of individuals consumed TFA above 1% of their total energy. Cheese was the main contributor to TFA intake, with processed/refined grains and fried fish following. The latter was the main contributor in older adults (51+ years). Adjusted logistic regression analysis showed that individuals at the highest tertile of trans consumption were 30% more likely to have dyslipidemia compared to the lowest (${\mathrm{OR}}_{\left(Q\,3-Q\,1\right)}$: 1.3; 95% CI: 1.02–1.66 and ${\mathrm{OR}}_{\left(Q\,2-Q\,1\right)}$: 1.3; 95% CI:1.01–1.66, respectively). This increased by 10% when stratified by SFA intake (OR: 1.4; 95% CI: 1.061–1.942) and remained significant only in individuals at the highest tertile and with higher than recommended SFA intake.

**Conclusions:**

A high intake of TFA combined with high SFA intakes further increase the likelihood of dyslipidemia and should be accounted for in public health prevention programs. Monitoring and evaluation of the recent EU legislative measures on TFA levels in foods is also necessary.

## 1. Introduction

Cardiovascular diseases (CVD) are a leading cause of death worldwide, including Europe [1, 2]. Ischemic heart disease and cerebrovascular disease have been the two leading causes of death in Greece during the past decade [2], mainly attributed to unfavorable changes in modifiable risk factors such as dyslipidemia [3]. Since the 1990s, accumulating and overwhelming evidence points to the detrimental effects of trans fatty acids (TFA) on human health, particularly with respect to cardiovascular health and total mortality [1, 4, 5, 6, 7].

Higher TFA intakes have been associated in general with a 20–30% increased risk of all-cause mortality, irrespective of replacement nutrients [8]. TFA are unsaturated fatty acids that contain at least one double bond in the trans configuration and can be of natural origin or industrially produced. The latter have been widely used in food manufacturing, such as bakery products and margarine, due to their increased plasticity and chemical stability. TFA, however, have been associated with adverse health effects, disrupting circulating lipid biomarkers; specifically increasing LDL-cholesterol, lipoprotein (little) a (Lpα) and triacylglycerol levels, decreasing HDL-cholesterol levels and LDL-cholesterol particle size [9, 10], but also increasing total-/HDL-cholesterol ratio [11]. TFA intake has also been shown to accentuate systemic inflammation, with a positive relationship being found between TFA intake and c-reactive protein (CRP) levels [12, 13], adversely affecting endothelial function. This may partially explain the higher than expected cardiovascular disease risk as a result of abnormal lipid profile [12].

Other to the direct effects of TFA’s to the cardiovascular system, they may also exert an indirect effect on it. Specifically, a linear association has been reported between higher TFA intake and increased weight gain and fat adiposity, as well as with impaired glucose tolerance [14, 15]. Based on the above adverse physiological effects, it has been reported that a 2% absolute energy intake from TFA is associated with a substantial increase in coronary heart disease (CHD) incidence, and specifically with 23% increase in CVD risk [16].

The World Health Organization (WHO) recommends that energy intake from TFA should not exceed 1%, including TFA of natural origin [17], and since 2015 it encourages TFA elimination in the food supply [18]. The European Food Safety Authority [19] also suggests that the intake of TFA should be as low as possible within the context of a nutritionally adequate diet. A study conducted between June 1995 and April 1996, assessed TFA intake in 14 Western European countries, one of which was Greece, and found that the population median of TFA consumption in Greece was among the lowest in Europe, ranging between 0.5% and 0.8% of total energy intake, for men and women respectively [20]. Since then a major transition has occurred towards a more Western type dietary pattern, with a simultaneous decreased adherence to a Mediterranean type diet [21]. Also, recently published [22] TFA concentration data of commonly consumed foods in Greece, indicated that certain foods can have TFA content exceeding 2% of total fat. It is therefore of great importance to acquire up-to-date information on total TFA intakes from a nationally representative sample of Greek adults.

Consequently, the aim of the present study was to conduct a TFA exposure assessment in Greek adults, identifying major contributing foods to this exposure and assess the association of TFA intake with likelihood of dyslipidemia and prevalence of other CVD risk factors, using a nationally representative sample.

## 2. Methods

### 2.1 Study design

This study included adults who were enrolled in the Hellenic National Nutrition and Health Survey (HNNHS), a population-based survey conducted between September 2013 and May 2015. The study was designed to assess the health and nutritional status of Greek residents, excluding individuals residing in institutions, members of the armed forces, pregnant and lactating women, and individuals with mental disabilities. Individuals were selected following a multi-stage stratified sampling design, by geographical region, area, sex, and age group. Study details have been published elsewhere [23]. A total of 3775 adults were enrolled in HNNHS and a total of 3537 individuals ≥19 years were included in this study (48.7% males) for which data on TFA intake were available. All work was carried out upon obtaining individual consent and approval by the Ethics Committee of the Department of Food Science and Human Nutrition of the Agricultural University of Athens and by the Hellenic Data Protection Authority (HDPA).

All individuals enrolled in the study were interviewed by trained personnel. An interviewer-administered questionnaire was used to obtain information on sociodemographics, anthropometric characteristics, medication intake, and lifestyle choices (such as smoking habits and level of physical activity).

### 2.2 Dietary & trans fatty acid assessment

Two 24 hr-recalls were collected; one during the first face-to-face interview, and the second through a telephone interview after 8–20 days on a different non-consecutive day, using the Automated Multiple Pass Method. For optimal intake assessment specific, validated food atlases and standardized household measures were used as portion anchors. The TFA content of the food groups used for this study was derived from two sources. The primary data source was the Nutrition Data System for Research (NDSR) developed by the University of Minnesota which is an integrated data system providing extended nutrient profile data [3] for globally consumed food. This system, however, does not contain ethnic consumed foods. Due to the high sensitivity requirements of TFA measurement, data from chemical analysis of 140 samples from different foods frequently consumed by the population residing in Greece, including fast food, pies and pastries, were used [22]. Details can be found in Appendix Table 3. To estimate the contribution of each food group (FG) to total TFA intake, foods reported in 24 hr were organized into 37FG’s, based on their composition (Appendix Table 4). Foods included in recipes/mixed dishes were assigned to multiple food groups according to the different foods that they consisted of and were then grouped as stated above.

The percentage of the contribution of each FG to TFA intake was derived by the following formula: % contribution of FG to TFA = (sum of TFA intake for that FG/sum of total TFA) * 100. This was calculated separately for each age and sex group. Total TFA intake (grams/day) was then transformed to total energy from TFA (TFA in grams * 9 kcal/per gram) and the latter was standardized by total mean energy consumption.

Data on total fat, SFA, Poly- and Mono- unsaturated fatty acids (PUFA & MUFA, respectively) and added sugars were also calculated as per total energy intake. Total fiber and cholesterol intake was measured in grams per day. Sodium from foods alone was also estimated and was reported in total grams per day.

### 2.3 Definition of dyslipidemia 

Individuals with dyslipidemia were defined as those reported having high plasma cholesterol and/or triglycerides levels, or on medication, or those who were classified as dyslipidemic based on the European Society of Cardiology cut-offs of lipid levels. These include (either/or): LDL-cholesterol ≥116 mg/dL; HDL-cholesterol ≤35 mg/dL in females and ≤40 mg/dL for males; total triglycerides >150 mg/dL; Total Cholesterol >200 mg/dL; or on antilipidemic medication. Therefore, those who were unaware of their status were also accounted for. The Friedewald Formula was used to calculate LDL-cholesterol [24] and since it is known that the Friedewald Formula is not sensitive for triglyceride values >400 mg/dL, individual data were checked and only 4 individuals (out of 1088) had such blood triglyceride levels.

LDL = Total Cholesterol – HDL-cholesterol – (TG/5), in mg/dL

(Where TG, Triglycerides; HDL, High Density Lipoprotein; LDL, Low Density Lipoprotein.)

All blood samples were collected in the morning, between 8:00 and 10:00 AM, after fasting for at least 10 hours. All biochemical examinations listed above, as well as fasting plasma glucose were carried out using enzymatic methods in Cobas Integra 400 analyzer (F. Hoffmann-La Roche Ltd., Basel, Switzerland).

### 2.4 Other parameters

BP measurements were taken with individuals rested for at least 5 minutes, seated with their back upright, and their arm well-supported at a ${\mathrm{45}}^{\circ }$ angle from the trunk at the heart level [25]. Three consecutive measurements taken on a single occasion were used to assess individuals blood pressure. The average of these measurements was used to describe and report the study populations mean SBP and DBP levels.

Sociodemographic and anthropometric data were collected by trained health professionals using Computer Assisted Personal Interview (CAPI) software. Specifics on age, sex and educational level were acquired by highly trained health professionals. Educational level was classified into 3 groups: <6 years of schooling; ≥6–11 years; and ≥12 years.

Smoking habits and physical activity level were also assessed. Individuals were classified as ex-smokers’ if they had stopped smoking at least for 30 days, smokers, or never-smokers. Physical activity (PA) was defined as light, moderate or high, according to the International Physical Activity Questionnaire (IPAQ), as per calculation guidelines [26]. Individuals scoring below the light activity level were categorized as sedentary. Weight (kg) and height (m) were measured from which Body Mass Index (BMI) was derived [${\mathrm{weight/height}}^{2}$ (kg/$m^{2}$)]. Weight status was categorized as “underweight <18.5 kg/$m^{2}$”, “18.5 ≤ normal weight < 25 kg/$m^{2}$”, “25 ≤ overweight < 30 kg/$m^{2}$”, and “obese ≥30 kg/$m^{2}$”.

### 2.5 Statistical analysis

Data were analyzed using appropriate methodology for survey design to have generalizable results to the reference population. Specifically, data were weighted by area, age group, and sex (as per sampling frame), using the 2011 Population Census. Continuous variables were presented as mean ± standard deviation (sd) when normally distributed, and median (IQR) for skewed distributions. Categorical variables were presented as frequencies with 95% confidence intervals (95% CI). CI’s were reported as informative for the population distribution, since this is a National representative study. Group differences were tested using chi square test for proportions, and ANOVA or Kruskal Wallis rank sum test for continuous data, depending on data distribution. *p* for trend was tested post hoc. Survey specific logistic regression model [with linearized SE’s] was used to assess the likelihood of dyslipidemia, by tertile of TFA intake. The model was adjusted for a priori known risk factors. Specifically, weight status, sex, age, smoking status, and sodium intake were introduced as categorical in the model, whereas saturated fat intake, physical activity level (IPAQ), educational level, and fiber intake as continuous. This was decided following a preliminary assessment on group differences. Logistic regression model was also stratified by SFA intake, to account for potential mediating effect between TFA and SFA intakes. All *p*-value estimates were based on two-sided tests. A *p*-value <0.05 was considered statistically significant. STATA 14.0 (StataCorp, Texas ltd., Texas, USA) statistical package was used for the analysis.

## 3. Results

The description of the main demographic, anthropometric dietary and other personal characteristics is depicted in Table 1. Overall, TFA intake did not differ by sex, age (in total and category), BMI, weight status and total energy consumption. Median TFA intake was 0.53% as energy (0.34% to 0.81%) in the total population but ranged from 0.27% in the first tertile to 0.95 in the 3rd, with a significant increasing trend (*p *< 0.001). A total of 16% of individuals consumed TFA above 1% of their total energy intake while a weighted 33.7% of the population had a TFA median intake of 0.95% of total energy, with an Interquartile range distribution of 0.81% to 1.31%. Individuals consuming highest TFA levels also had significant higher intakes of total fat, SFA, PUFA and MUFA (all expressed as % of total energy intake). Large differences were observed in SFA intake with individuals at the 1st tertile (Q1) of TFA intake consuming on average 10%, individuals at the 2nd TFA tertile consuming 13% (Q2) and those at the 3rd, 14.6% 3rd (Q3). This increasing trend was also observed in total cholesterol and sodium intakes (*p *< 0.001 for between group differences and for trend). Total fiber intake was significantly lower in the highest tertile of TFA intake with a significant decreasing trend found (*p *< 0.001). Mean systolic and diastolic blood pressures (SBP and DBP respectively), smoking, marital and professional status, as well as educational level, did not significantly differ.

**Table 1.** **Distribution of demographic, anthropometric, dietary, and other personal characteristics of the HNNHS population in total and by tertile of TFA intake**.

Variables		Tertile of Trans fatty acid intake				
	Total Population N = 3537	1st Tertile N = 1163	2nd Tertile N = 1196	3rd Tertile N = 1178	*p* for differences	*p* for trend
Trans fatty acid intake, as % energy		0.27 (0.1, 0.34)	0.53 (0.47, 0.61)	0.95 (0.81, 1.31)	<0.001	<0.001
Sex, % (95% CI)						
Males	48.7%	50.4 (47.7, 53.1)	47.2 (44.3, 50.2)	47.4 (44.5, 50.2)	0.26	
Age in years, mean (sd)		44.1 (18.5)	42.9 (18.3)	43.8 (19.1)	0.202	0.499
Age category, % (95% CI)					0.254	
18–39 years	40.0 (45.1, 48.9)	31.1 (28.7, 33.6)	35.9 (33.5, 38.5)	33.0 (30.6, 35.5)		
40–59 years	32.1 (30.4, 33.9)	37.3 (34.1, 40.6)	30.0 (27.0, 33.1)	32.7 (29.6, 36.0)		
≥60 years	20.9 (19.2, 22.7)	31.3 (27.2, 35.8)	32.1 (28.2 (36.3)	36.5 (32.3, 41.1)		
BMI (kg/$m^{2}$)	25.5 (4.7)	25.6 (4.8)	25.5 (4.8)	25.3 (4.6)	0.816	0.161
BMI category, % (95% CI)					0.176	0.358
Healthy weight	88.2 (46.3, 50.1)	47.8 (44.6, 51.1)	37.2 (33.9, 40.4)	15.1 (12.9, 17.6)		
Overweight	34.7 (32.9, 36.6)	47.8 (44.7, 51.2)	33.1 (30.1, 36.3)	18.9 (16.4, 21.8)		
Obesity	17.1 (15.6, 18.7)	48.8 (45.6, 52.1)	33.9 (31.0, 37.0)	17.3 (14.8, 20.0)		
Total energy in kcals, mean (sd)	1937 (859)	1956 (904)	1915 (817)	1942 (856)	0.022	0.501
Total fat, % energy, mean (sd)	38.1 (10.3)	35.0 (11.9)	38.1 (9.3)	41.0 (8.7)	<0.001	<0.001
Trans fat, % energy, median, IQR	0.53 (0.34, 0.81)	0.27 (0.17, 0.34)	0.53 (0.47, 0.61)	0.95 (0.81, 1.31)	<0.001	<0.001
SFA, % energy, mean (sd)	12.6 (4.3)	10.1 (3.8)	13.0 (3.6)	14.6 (4.3)	<0.001	<0.001
PUFA, % energy, median IQR	4.9 (3.9, 6.4)	4.8 (3.7, 6.3)	4.8 (3.8, 6.3)	5.2 (4.1, 6.5)	<0.001	<0.001
MUFA, % energy, mean (sd)	17.1 (6.1)	16.9 (7.3)	16.7 (5.6)	17.6 (5.2)	0.003	<0.001
Added sugars, % energy, median IQR	9.9 (4.9, 16.8)	8.9 (3.9, 15.8)	10.6 (5.3, 17.4)	10.3 (5.6, 17.2)	0.232	
Fiber (gr), median IQR	18.4 (12.1, 33.9)	22.6 (13.9, 47.8)	18.1 (12.3, 31.3)	14.3 (6.1, 22.2)	<0.001	<0.001
Cholesterol (gr), median IQR	203 (126, 313)	162 (90, 274)	202 (133, 305)	238 (158, 360)	<0.001	<0.001
Total sodium (mg), mean (sd)	2087 (738)	1927 (737)	2109 (702)	2222 (746)	<0.001	<0.001
Total METS, median IQR	2226 (984, 4986)	2466 (990, 5280)	2160 (942, 4746)	2148 (990, 4764)	0.242	0.201
Smoking status, % (95% CI)					0.633	-
Ex-smoker	16.8 (15.4, 18.3)	33.6 (29.2, 38.2)	33.6 (29.4, 38.2)	32.8 (28.5, 37.4)		
Current smoker	34.2 (32.3, 36.2)	34.1 (31.0, 37.3)	33.9 (30.8, 37.0)	32.1 (29.1, 35.3)		
Never smoker	49.0 (47.0, 51.0)	31.5 (28.9, 34.2)	33.5 (31.0, 36.2)	34.9 (32.3, 37.6)		
Systolic BP in mmHg, mean	118.6 (15.3)	119.1 (14.4)	117.3 (15.5)	119.5 (15.8)	0.754	0.804
Diastolic BP in mmHg, mean	72.0 (10.6)	71.5 (10.8)	72.1 (11.0)	72.5 (10.0)	0.422	0.276
Dyslipidemia, % (95% CI)	27.6 (26.0, 29.3)	28.0 (23.3, 28.9)	28.2 (25.4, 31.1)	28.7 (25.8, 31.8)	0.393	-
Professional status, % (95% CI)					0.232	-
Employed	49.1 (47.1, 51.2)	31.3 (28.7, 34.0)	35.6 (32.9, 38.3)	33.1 (30.6, 35.9)		
Unemployed	20.0 (18.5, 21.5)	35.6 (31.7, 39.7)	32.7 (29.0, 36.6)	31.7 (28.0, 35.6)		
Homeworkers	7.7 (6.8, 8.7)	33.9 (27.8, 40.5)	28.4 (22.8, 34.9)	37.7 (31.3, 44.5)		
Pensioners	23.2 (21.4, 25.0)	32.4 (28.4, 36.6)	32.2 (28.5, 36.2)	35.4 (31.3, 39.7)		
Educational Level in school years, % (95% CI)					0.383	-
<6	14.9 (13.3, 16.6)	36.0 (30.6, 41.7)	31.5 (26.6, 36.8)	32.5 (27.5, 38.0)		
≥6–11	35.9 (34.0, 37.8)	33.9 (30.9, 37.1)	32.9 (29.9, 36.0)	33.2 (30.2, 36.4)		
≥12	49.2 (47.2, 51.3)	30.9 (28.5, 33.5)	34.9 (32.4, 37.3)	34.2 (31.7, 36.8)		
Marital status, % (95% CI)						
Single/Divorced/Separated	44.1 (42.1, 46.1)	31.7 (29.1, 34.3)	34.7 (32.1, 37.4)	33.6 (31.1, 36.3)	0.692	-
Widowed	7.5 (6.5, 8.7)	32.5 (26.2, 39.4)	30.6 (24.3, 37.7)	36.9 (30.2, 44.2)		
Married/Cohabitation agreement	48.4 (46.3, 50.5)	33.5 (30.6, 36.5)	33.5 (30.8, 36.2)	33.0 (30.2, 35.9)		

All proportions are weighted by area, sex and age. Group differences were tested using chi square test for proportions, and ANOVA or Kruskal Wallis rank sum test for continuous data, depending on data distribution. *p* for trend was tested post hoc.

**Fig. 1.**
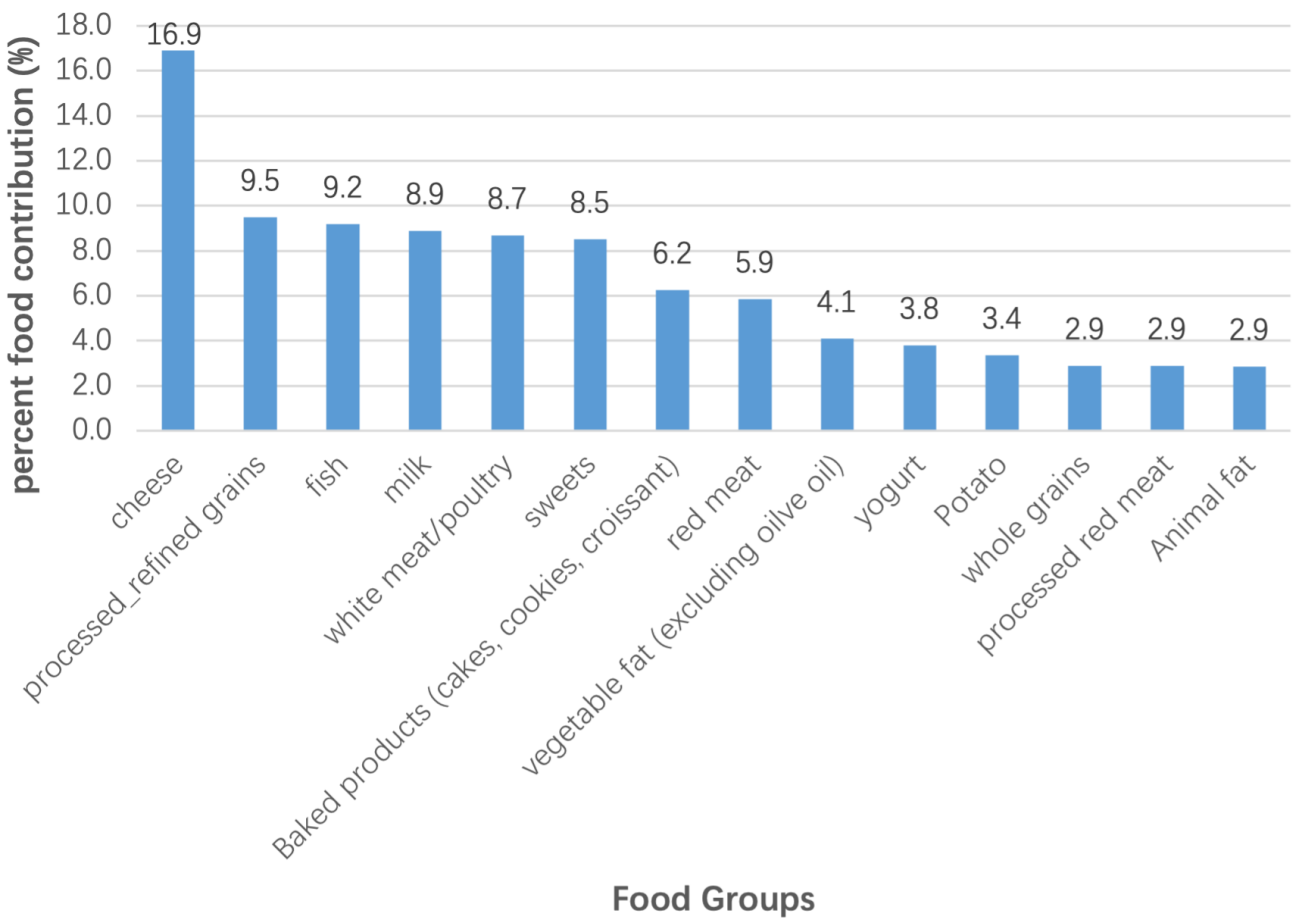
**Main food group contribution to TFA in total population**. The three most contributing food groups to TFA intake in adults in Greece are cheese, processed/refined grains such as pies/pastries and fried fish.

In Fig. 1 the main food groups that contribute to TFA intake are depicted, including dairy and meat (poultry and red), in which TFA are found naturally. Cheese was by far the main contributor to TFA intake, with processed/refined grains and fried fish following.

**Fig. 2.**
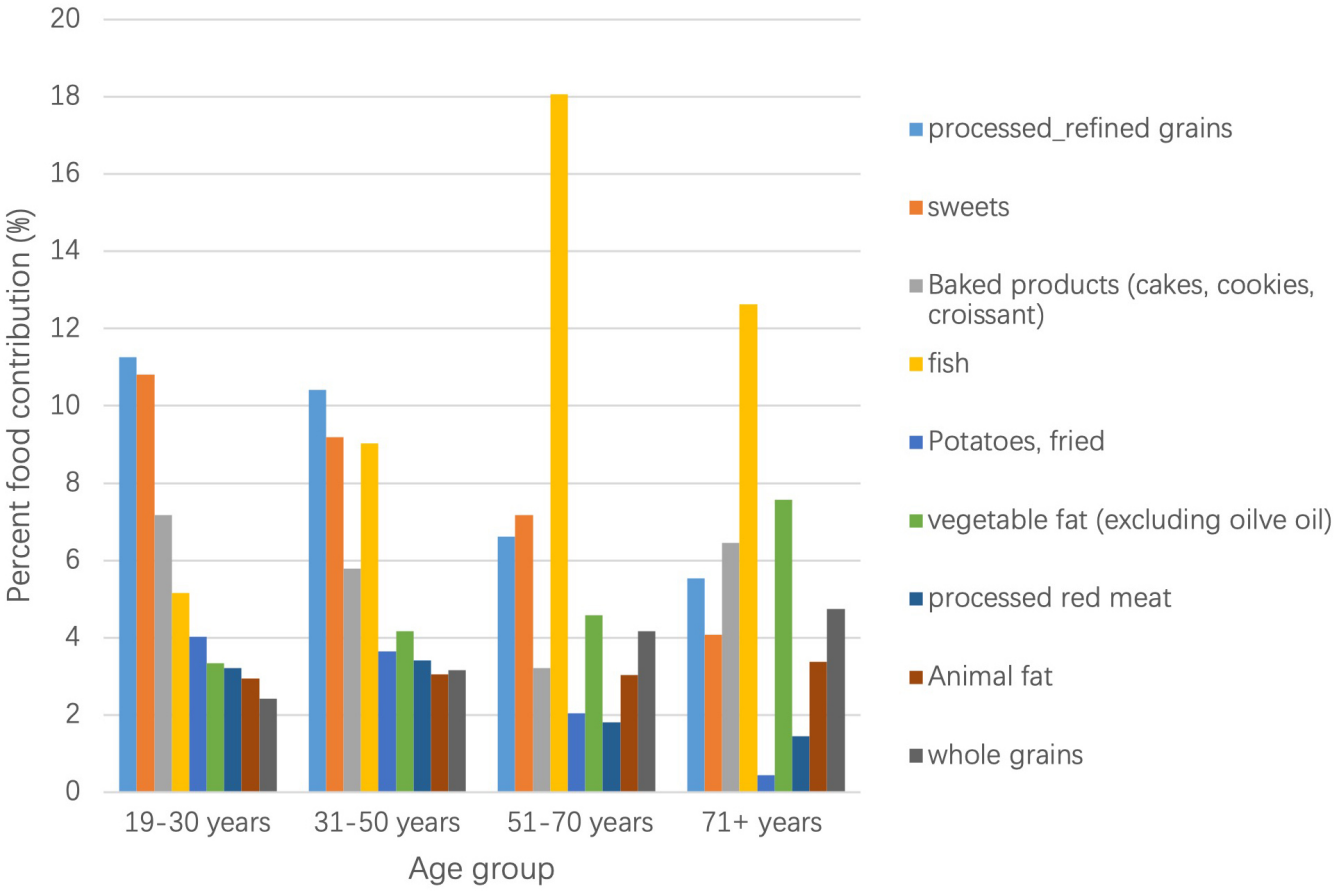
**Main food group contribution to TFA by age group**. The most contributing food group to TFA intake (excluding naturally ocurring TFA) for the age groups 19–30 y and 31–50 y was processed/refined grains, while for the age groups 51–70 y and 71+ y this was fried fish.

In Fig. 2 main food contributors by age group are presented, in this case excluding food groups with naturally occurring TFA’s. Although total TFA intake did not differ by age group, the weight of the major contributing foods highly differed. Specifically, processed refined grains (mainly from savory pastries & pies) and sweets were the main food contributors in younger adults, whereas fried fish clearly picked in older adults, with a 13% contribution in adults 71+ years and 18% in those between 51 and 70 years.

**Fig. 3.**
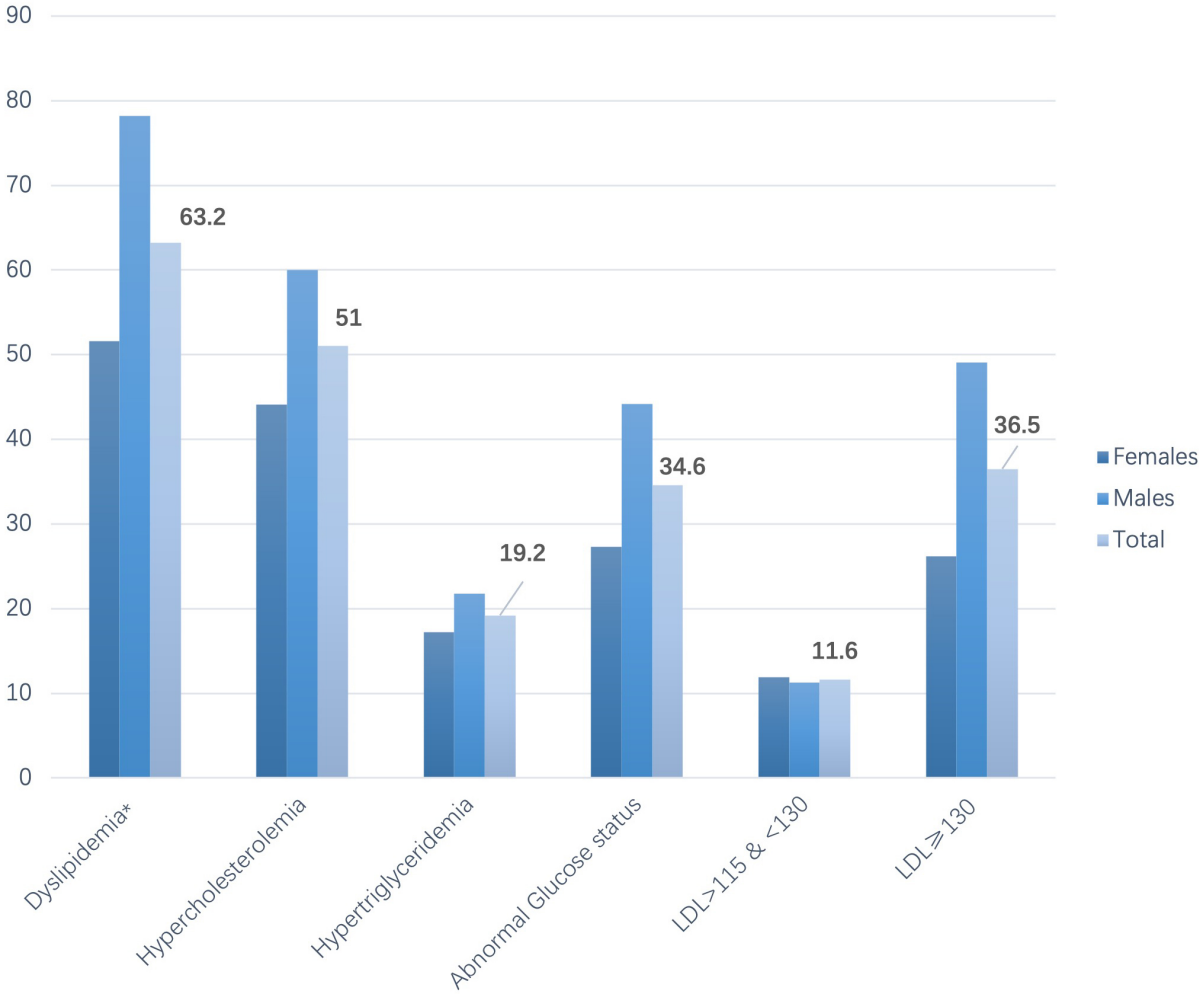
**Proportion of population with specific disease status that consume over 1% of total energy from trans-fat, in total and by sex (N = 577, 46.35% of population)**. LDL, Low density Lipoprotein, all in md/dL. Dyslipidemia as per measured abnormal lipid profile. Hypercholesterolemia: >200 mg/dL. Abnormal Glucose status: Fasting plasma glucose >110 mg/dL.

A descriptive presentation of the proportion of the population with specific CVD risk factors among those consuming above the recommended levels of TFA intake (>1% of total energy) for the total population and by sex is presented in Fig. 3. Overall hypercholesterolemia and total dyslipidemia affected more than 50% of the population with TFA consumption above recommended intakes, with prevalence being significantly higher in males, although total distribution intakes did not differ (as per Table 1). Clinically significant prevalence was also observed in those with abnormal plasma glucose (>110 mg/dL) and LDL-cholesterol levels (≥130 mg/dL).

Dyslipidemia likelihood in total and by level of SFA intake is shown in Table 2. A fully adjusted model, accounting for weight status saturated fat intake, sex, age, smoking status, physical activity level (IPAQ), educational level, and fiber intake, showed that dyslipidemia was 30% more likely for those at the 2nd tertile or the 3rd tertile compared to the lowest intakes (OR: 1.3; 95% CI: 1.02–1.66 and 1.01–1.66, respectively). When the logistic regression was stratified by SFA intake above and below recommended guidelines (10% of total energy), the likelihood of dyslipidemia increased to 40%, (OR: 1.4; 95% CI: 1.06–1.94). The results remained significantly higher only among those with >10% SFA intakes. Higher physical activity, and never smoking significantly reduced the odds of dyslipidemia but did not null the risk attributed to higher TFA and SFA intakes. Overweight and obesity as well as increasing age categories significantly increased the odds of dyslipidemia in all cases.

**Table 2.** **Likelihood of Dyslipidemia by tertile intake of trans fatty acids, in total and by level of saturated fat intake**.

Dyslipidemia				<10% Saturated fats			≥10% Saturated fat		
	Odds Ratio	Std. Err.	[95% Conf. Interval]	Odds Ratio	Std. Err.	[95% Conf. Interval]	Odds Ratio	Std. Err.	[95% Conf. Interval]
Trans intake % energy 1st Tertile the reference level									
2nd tertile	1.3	0.2	1.02, 1.66	1.4	0.3	0.94, 2.22	1.3	0.2	0.97, 1.80
3rd tertile	1.3	0.2	1.01, 1.66	0.8	0.2	0.49, 1.34	1.4	0.2	1.06, 1.94
Weight ${\mathrm{status}}^{1}$	1.7	0.2	1.44, 2.11	2.0	0.4	1.38, 2.80	1.7	0.2	1.34, 2.11
${\mathrm{Sex}}^{2}$	1.1	0.1	0.87, 1.27	1.0	0.2	0.70, 1.40	1.1	0.1	0.85, 1.35
Age ${\mathrm{category}}^{3}$									
40–59	3.7	0.4	2.93, 4.51	5.0	1.1	3.23, 7.66	3.3	0.4	2.60, 4.29
≥60	5.4	0.9	3.70, 7.02	7.4	2.3	3.98, 13.72	4.6	0.9	3.20, 6.68
Smoking ${\mathrm{status}}^{4}$									
current	0.8	0.1	0.66, 1.13	0.9	0.2	0.56, 1.52	0.9	0.1	0.62, 1.18
never	0.7	0.1	0.52, 0.86	0.8	0.2	0.47, 1.26	0.6	0.1	0.48, 0.85
Physical activity ${\mathrm{level}}^{5}$	0.7	0.1	0.52, 0.81	0.5	0.1	0.35, 0.83	0.7	0.1	0.53, 0.88
Educational ${\mathrm{level}}^{5}$	1.1	0.1	0.95, 1.30	1.2	0.2	0.88, 1.52	1.1	0.1	0.92, 1.32
Total Saturated fat intake, % energy	0.9	0.1	0.70, 1.11	-	-	-	-	-	-
Sodium intake (>1500 compared to <1500)	0.9	0.1	0.79, 1.05	1.0	0.1	0.74, 1.231	0.9	0.1	0.75, 1.07
Total MUFA intake, % energy	1.0	0.0	0.99, 1.02	1.0	0.0	0.99, 1.05	1.0	0.0	0.98, 1.02

Reference categorization: ^1^overweight & obesity vs healthy weight; ^2^baseline level 19–39.9; ^3^females compared to males; ^4^compared to ex-smokers; ^5^assessed as continuous variables. model adjusted for weight status, saturated fat intake, sex, age, smoking status, physical activity level (IPAQ), educational level, and fiber intake. MUFA, Monounsaturated fatty acids.

## 4. Discussion 

The present study showed that higher TFA intakes were significantly associated with an increased likelihood of dyslipidemia with prevalence of dyslipidemia reaching 63% among adults that consumed TFA above the recommended intake which is set to 1% of total energy intake. Also, although approximately 16% of the population exceeded the recommended levels of TFA intake, the median intake of the population at the highest tertile was 0.95% This means that approximately 1/3 of the population had an intake borderline to the recommended cut-off level. This proportion of the population also greatly exceeded SFA recommended intakes by 4.6%, a factor that showed to further increase likelihood of dyslipidemia by an additional 10%. The major food groups contributing to TFA intakes were a mix of natural and industrially produced TFA’s, with an emphasis on cheese, processed grains, fried fish, and baked goods & sweets. These results are in agreement with other studies [27] who reported a higher CVD risk with increasing TFA intakes and underline the need for public health prevention programs.

In 2020 WHO created a Certification Programme for Trans Fat Elimination in order to recognize countries that have eliminated industrially produced TFA’s, with 14 countries, including USA, being recognized with best-practice and monitoring and enforcement systems in place already in effect policies [18]. TFA’s can be of natural (ruminant) origin (r-TFA) or industrially produced (i-TFA). Industrial sources of TFA are mainly of concern, since the consumption of r-TFA on average contributes to less than 0.5% of total energy intake [14]. Recently, the EU Regulation 2019/649 (EC, 2019) set a limit of 2% i-TFA per 100 g of fat in processed foods, in an effort towards further i-TFA reduction in the food supply in the EU, a regulation fully implemented since April 2021. A reduction of i-TFA intake has been a global public health priority. However, the question remains whether a banning policy alone will effectively decrease CVD risk, since the present study revealed that even levels close to but below 1% TFA intake with respect to energy consumption are associated with increased likelihood of dyslipidemia, especially among individuals that have SFA intakes >10% of their total energy consumption. This is of major importance since studies that have evaluated implemented mandatory TFA limit policy, showed that in foods that had decreased their TFA content to adequate levels, in some cases SFA content increased [28, 29, 30, 31], while in other cases unsaturated fats increased [32]. Of course, it should be mentioned that a recent meta-analysis of epidemiological studies did not find a significant increased risk of CVD outcomes with SFA intake, but it was associated with TFA [8]. The studies included however, did not assess TFA intake by level of SFA consumption, hence the results are not comparable. Mazidi *et al*. [33], reported that SFA intake was associated with all-cause mortality in the National Health and Nutrition Examination Survey (NHANES). When the authors performed a meta-analysis on end point associated with SFA intake they found a significant association with CHD only [33]. Another study showed that non-optimal SFA and TFA intakes accounted for 3.6% and 7.7% of global CHD mortality, with important between country heterogeneity [7]. The type of fat consumed, may therefore, affect health outcome [7] and may also be population specific based on dietary, lifestyle and other variables.

In the present study r-TFA’s were not distinguished from i-TFA’s since a recent systematic review reported that both sources of TFA can increase cardiometabolic risk parameters, especially lipid profile [6]. Specifically, although rTFA seems to be less harmful than iTFA for HDL cholesterol, in the case of total cholesterol and LDL cholesterol it may be worse. This is of great importance considering that LDL is one of the strongest determinants of CVD risk and high levels of LDL were found in 36.5% of this study’s population that consumed TFA >1% of their total energy. In addition, considering the potential mediating effect of SFA on dyslipidemia, the fact that cheese was the main food item contributing to TFA with an approximately 7% marked difference compared to the 2nd main contributor (refined/processed grains) raises concerns on the effectiveness of the implemented TFA policy, if educational and other promotional campaigns are not administered.

TFA’s are also present in baked and fried foods and significantly intake in the Greek population. Interestingly, results from the present representative study showed that apart from cheese, another major contributor to total TFA food was processed grains and their products and in particular baked goods such as savory and sweet pastries and pies.

Finally, the third major food which contributed highly to total TFA intake was fried fish, in all age groups, and primarily in adults over 50 years of age, indicating that the method of cooking could also significantly contribute to total TFA intake. This is of special interest since fish is a food that is perceived by most individuals as healthy and can be consumed in restaurants or at home. Since there are no available occurrence data on TFA content of fried fish prepared at home and/or out of home in Greece, these results should be viewed with caution and point to a need to focus on sampling fried fish, particularly from the catering sector, in future official food controls in Greece.

The results of this large, national cross-sectional study have been presented with caution due to the nature of the design and should be treated accordingly. Specifically, only the likelihood of outcomes can be evaluated with respect to specific risk factors, in this case dyslipidemia and level of TFA intake, and no temporal effects can be established. Other strong end points were not included, such as CVD outcomes since people tend to change their dietary and lifestyle habits after a specific event. This would have included systematic exposure measurement error in the analysis. The study however also has strong points, since it is a strategically designed study that aimed to evaluate the nutritional and health status using a national representative sample. Furthermore, TFA analysis was performed using country specific data, particularly for baked goods consumed, obtained during an official control program by the Hellenic Food Authority.

## 5. Conclusions

Dyslipidemia prevalence increased with higher total TFA intake, especially among those with high SFA intakes, underlining the need for stricter adherence to dietary guidelines following educational programs along with set public health policies. These are both highly modifiable factors and can greatly serve as vehicles to reduce dyslipidemia, a major cardiovascular risk factor. Both r-TFA and i-TFA should be monitored and further evaluated by level of SFA intake. Although i-TFA is expected to decrease following the implemented TFA elimination policy, monitoring the lipid profile of processed foods, particularly non-branded/non-prepackaged foods such as bakery foods and fried fish, and checking the abidance of the food and catering sector to the new EU legislation on i-TFA is necessary and important.

## Abbreviations

TFA, Trans Fatty Acids; Itfa, industrialized Trans Fatty Acids; rTFA, ruminant Trans Fatty acids; PUFA, Polyunsaturated fatty Acids; MUFA, Mono unsaturated Fatty acids; SFA, Saturated Fatty Acids; BMI, Body Mass Index; FG, Food groups; HNNHS, Hellenic National Nutritiona and Helath Survey; EU, European Union; CVD, Cardiovascular Diseae; CRP, C Reacive Protein; HDL, High Density Lipoprotein; LDL, Low Density Lipoprotein; Lpα, lipoprotein (little) a; TG, Triglycerides; PA, Physica activity; WHO, World Health Organization; IPAQ, International Physical Activity Questionnaire; CI, Confidence Interval.

## Appendix

*TFA Content of Foods*

Food items sampled and analyzed are shown in Table 3. The mean TFA from multiple measurements was calculated and used for ethnically consumed food.

The TFA was recalculated for 5557 out of 87953 food consumed by the HNNHS sample. representing a percentage of 6.3% and for 97 out of 1915 unique foods (5.1%).

**Table 3.** **Content of Trans Fatty Acids (g/100g food). total (TFA) and industrial (i-TFA) per food group**.

Food Groups			Samples analyzed	HNNHS (n)	HNNS unique foods list (n)	TFA g/100 g food	i-TFA g/100 g food
Savoury (i.e., non-sweet) foods and snacks			70	2703	39		
	Cheese pies		30	712	6	0.58	0.49
		Cheese pies kaseropita	2	14	2	0.87	0.72
	Spinach pies		5	235	4	0.09	0.09
	Sausage pies		5	67	3	0.43	0.40
	Pizza		12	451	3	0.16	0.01
		Pizza tomato. cheese margarita	4	55	2	0.21	0.04
	Meat products		10	1094	15		
		Pork skewers	3	265	3	0.05	0.05
		Pork gyros	3	201	4	0.09	0.09
		Chicken gyros	1	88	3	0.11	0.11
		Βurger (no bread) bifteki	1	498	1	0.08	0.08
		Kebab	2	42	4	0.26	0.18
	French Fries		5	44	7	0.05	0.05
	Pop corn		3	100	1	0.12	0.12
Dessert/sweet foods and snacks			70	2854	58		
	Cakes		20	608	21	0.09	0.07
		Cakes	10	531	15		
	Cake vanilla-chocolate/cocoa		2	22	1	0.08	0.08
	Cake cocoa		2	23	1	0.05	0.05
		Gateaux type layer cakes	10	77	6	0.2	0.00
	Gateaux cake almond		2	6	1	0.172	0.00
	Gateaux cake vanilla-chocolate		2	9	1	0.322	0.00
	Gateaux cake chocolate		1	33	1	0.28	0.00
	Cookies/biscuits		15	1113	15		
		Cookies	10	940	12	0.23	0.13
	Butter cookies		1	55	1	0.21	0.00
	Cinnamon cookies		2	58	1	0.07	0.03
	Grape must cookies		1	79	1	0.08	0.08
	Chocolate/cocoa cookies		1	41	1	0.11	0.10
		Biscuits	5	173	3	0.07	0.07
	Stuffed biscuits		4	169	1	0.08	0.08
	Croissants		10	382	8	0.18	0.07
	Doughnuts		10	112	2		
		Doughnuts Loukoumas	5	53	1	0.07	0.04
		Doughnuts Donuts	5	59	1	0.08	0.08
	Sweet Pastries (Bougatsa)		5	54	1	0.48	0.47
	Wafers		5	104	3	0.61	0.59
	Ice creams		5	481	8	0.13	0.01
		Ice cream parfait	1	9	1	0.14	0.00
Sum			140	5557	97		

**Table 4.** **Total Food Groups used for TFA study analysis**.

Food Groups		
**Fruits**	**Egg**	**Artificially-sweetened beverages**
Fresh fruits, cooked or dried	Eggs	Carbonated artificially-sweetened beverages
**Fruit juice, 100%**	**Fish and Shellfish**	**Salty snacks**
Natural fruit juices unsweetened	Fish fresh and frozen	Chips, crackers, pop corn
**Non-starchy vegetables**	Shellfish	**Desserts and Sweets**
Green leafy vegetables	**Red meat**	Sweets, candy, chocolate
Tomatoes, carrots, lettuce	Lamb, pork, veal, game	Milk desserts
Mixed and other vegetables	**White meat**	Sugary foods (i.e., baklavas)
Vegetable juice	Poultry	**Condiments and spices**
**Starchy vegetables**	**Processed meat**	**Salt all types**
Corn, beans, green beans	Sausages, ham, salami, beacon of red meat origin	**Water from mixed recipes**
Pumpkin	**Processed white meat**	Water natural, mineral and carbonated
Sweet potatoes	Sausages of white meat origin	**Coffee**
**Potato**	Chicken nuggets	**Tea**
Potatoes	**Processed fish**	**Artificially sweeteners**
**Wholegrain cereals**	Smoked, caned and salted fish	**Sugar**
Wholegrain cereal products	Fish sticks	Sugar, honey, syrup
**Processed cereals**	**Olive oil and olives**	**Baked products**
All refined grains and cereal products	Olive oils	Cake, biscuit, pie, muffin, doughnuts
**Legumes**	Oils	**Artificially sweetened Fruit juices**
Legumes, (i.e., beans)	**Vegetable fat**	Artificially sweetened fruit juices
Meat alternatives, soy, tofu	Vegetable oils, vegetable fat, vegetable oil-based salad dressing	**Baby food**
**Nuts**	**Animal fat**	Baby food
Nuts, almonds, seeds	Butter, mayonnaise	
Peanut butter	White sauce, cream	
Almond milk	Alcoholic beverages	
**Milk**	Alcoholic beverages	
Milk and milk drinks	**Sugar-sweetened beverages**	
**Yogurt**	Carbonated and non-carbonated sugar-sweetened beverages	
Yogurt		
**Cheese**		

## References

[1] (2015). ESC: Trans Fats Not Safe for Consumption.

[2] (2019). What causes the most deaths. http://www.healthdata.org/greece.

[3] Michas G, Karvelas G, Trikas A (2019). Cardiovascular disease in Greece; the latest evidence on risk factors. *Hellenic Journal of Cardiology*.

[4] Federici C, Detzel P, Petracca F, Dainelli L, Fattore G (2019). The impact of food reformulation on nutrient intakes and health, a systematic review of modelling studies. *BMC Nutrition*.

[5] Menaa F, Menaa A, Menaa B, Tréton J (2013). Trans-fatty acids, dangerous bonds for health? A background review paper of their use, consumption, health implications and regulation in France. *European Journal of Nutrition*.

[6] Verneque BJF, Machado AM, de Abreu Silva L, Lopes ACS, Duarte CK (2020). Ruminant and industrial trans-fatty acids consumption and cardiometabolic risk markers: a systematic review. *Critical Reviews in Food Science and Nutrition*.

[7] Wang DD, Li Y, Chiuve SE, Stampfer MJ, Manson JE, Rimm EB (2016). Association of Specific Dietary Fats with Total and Cause-Specific Mortality. *JAMA Internal Medicine*.

[8] de Souza RJ, Mente A, Maroleanu A, Cozma AI, Ha V, Kishibe T (2015). Intake of saturated and trans unsaturated fatty acids and risk of all cause mortality, cardiovascular disease, and type 2 diabetes: systematic review and meta-analysis of observational studies. *British Medical Journal*.

[9] Mozaffarian D, Clarke R (2009). Quantitative effects on cardiovascular risk factors and coronary heart disease risk of replacing partially hydrogenated vegetable oils with other fats and oils. *European Journal of Clinical Nutrition*.

[10] Wang Y, Proctor SD (2013). Current issues surrounding the definition of trans-fatty acids: implications for health, industry and food labels. *The British Journal of Nutrition*.

[11] Micha R, Mozaffarian D (2009). Trans fatty acids: effects on metabolic syndrome, heart disease and diabetes. *Nature Reviews Endocrinology*.

[12] Lopez-Garcia E, Schulze MB, Meigs JB, Manson JE, Rifai N, Stampfer MJ (2005). Consumption of trans fatty acids is related to plasma biomarkers of inflammation and endothelial dysfunction. *The Journal of Nutrition*.

[13] Mozaffarian D, Pischon T, Hankinson SE, Rifai N, Joshipura K, Willett WC (2004). Dietary intake of trans fatty acids and systemic inflammation in women. *The American Journal of Clinical Nutrition*.

[14] Micha R, Mozaffarian D (2008). Trans fatty acids: Effects on cardiometabolic health and implications for policy. *Prostaglandins, Leukotrienes and Essential Fatty Acids*.

[15] Barnard ND, Bunner AE, Agarwal U (2014). Saturated and trans fats and dementia: a systematic review. *Neurobiology of Aging*.

[16] Islam MA, Amin MN, Siddiqui SA, Hossain MP, Sultana F, Kabir MR (2019). Trans fatty acids and lipid profile: a serious risk factor to cardiovascular disease, cancer and diabetes. *Diabetes & Metabolic Syndrome: Clinical Research & Reviews*.

[17] Nishida C, Uauy R, Kumanyika S, Shetty P (2004). The joint who/FAO expert consultation on diet, nutrition and the prevention of chronic diseases: process, product and policy implications. *Public Health Nutrition*.

[18] WHO announces certification programme for trans fat elimination. https://www.who.int/news/item/17-11-2020-who-announces-certification-programme-for-trans-fat-elimination#:~:text=A%20new%20WHO%20Certification%20Programme,from%20their%20national%20food%20supplies.

[19] EFSA: Panel on Dietetic Products, Nutrition and Allergies (NDA) (2010). Scientific Opinion on Dietary Reference Values for fats, including saturated fatty acids, polyunsaturated fatty acids, monounsaturated fatty acids, trans fatty acids, and cholesterol. *EFSA Journal*.

[20] Hulshof KF, van Erp-Baart MA, Anttolainen M, Becker W, Church SM, Couet C (1999). Intake of fatty acids in western Europe with emphasis on trans fatty acids: the TRANSFAIR Study. *European Journal of Clinical Nutrition*.

[21] Kyriacou A, Evans JMM, Economides N, Kyriacou A (2015). Adherence to the Mediterranean diet by the Greek and Cypriot population: a systematic review. *European Journal of Public Health*.

[22] Marakis G, Fotakis C, Tsigarida E, Mila S, Palilis L, Skoulika S (2020). Fatty acid profile of processed foods in Greece with focus on trans fatty acids. *Journal of Consumer Protection and Food Safety*.

[23] Magriplis E, Dimakopoulos I, Karageorgou D, Mitsopoulou A, Bakogianni I, Micha R (2019). Aims, design and preliminary findings of the Hellenic National Nutrition and Health Survey (HNNHS). *BMC Medical Research Methodology*.

[24] Friedewald WT, Levy RI, Fredrickson DS (1972). Estimation of the Concentration of Low-Density Lipoprotein Cholesterol in Plasma, without Use of the Preparative Ultracentrifuge. *Clinical Chemistry*.

[25] Whelton PK, Carey RM, Aronow WS, Casey DE, Collins KJ, Dennison Himmelfarb C (2018). 2017 ACC/AHA/AAPA/ABC/ACPM/AGS/APhA/ASH/ASPC/NMA/PCNA Guideline for the Prevention, Detection, Evaluation, and Management of High Blood Pressure in Adults. A Report of the American College of Cardiology/American Heart Association Task Force on Clinical Practice Guidelines. *Hypertension*.

[26] Sjostrom M, Ainsworth BE, Bauman A, Bull FC, Hamilton-Craig CR, Sallis JF (2005). Guidelines for data processing analysis of the International Physical Activity Questionnaire (IPAQ) - Short and long forms. https://www.semanticscholar.org/paper/Guidelines-for-data-processing-analysis-of-the-and-Sjostrom-Ainsworth/efb9575f5c957b73c640f00950982e618e31a7be.

[27] Zhu Y, Bo Y, Liu Y (2019). Dietary total fat, fatty acids intake, and risk of cardiovascular disease: a dose-response meta-analysis of cohort studies. *Lipids in Health and Disease*.

[28] Downs SM, Thow AM, Leeder SR (2013). The effectiveness of policies for reducing dietary trans fat: a systematic review of the evidence. *Bulletin of the World Health Organization*.

[29] Mozaffarian D, Stampfer MJ (2010). Removing industrial trans fat from foods. *British Medical Journal*.

[30] Mozaffarian D, Jacobson MF, Greenstein JS (2010). Food reformulations to reduce trans fatty acids. *The New England Journal of Medicine*.

[31] Van Camp D, Hooker NH, Lin CJ (2012). Changes in fat contents of us snack foods in response to mandatory trans fat labelling. *Public Health Nutrition*.

[32] Ratnayake WMN, L’Abbe MR, Mozaffarian D (2009). Nationwide product reformulations to reduce trans fatty acids in Canada: when trans fat goes out, what goes in. *European Journal of Clinical Nutrition*.

[33] Mazidi M, Mikhailidis DP, Sattar N, Toth PP, Judd S, Blaha MJ (2020). Association of types of dietary fats and all-cause and cause-specific mortality: a prospective cohort study and meta-analysis of prospective studies with 1,164,029 participants. *Clinical Nutrition*.

